# Supplementary material for: Methylated Circulating Tumor DNA in Blood as a Tool for Diagnosing Lung Cancer: A Systematic Review and Meta-Analysis
Source: Cancers (Basel). 2023 Aug 3;15(15):3959. doi: 10.3390/cancers15153959 (PMC10417522; doi:10.3390/cancers15153959)

Supplementary Materials: Methylated circulating tumor DNA in blood as a tool for diagnosing lung cancer: A systematic review and meta-analysis

Morten Borg [1,#], Sara Witting Christensen Wen [2,3, #,*], Rikke Fredslund Andersen [4], Signe Timm [2,3], Torben Frøstrup Hansen [2,3], and Ole Hilberg [1,3]

[1] Department of Medicine, Vejle Hospital, University Hospital of Southern Denmark; morten.hornemann.borg@rsyd.dk
[2] Department of Oncology, Vejle Hospital, University Hospital of Southern Denmark; sara.witting.christensen.wen@rsyd.dk
[3] Department of Regional Health Research, University of Southern Denmark
[4] Department of Biochemistry and Immunology, Vejle Hospital, University Hospital of Southern Denmark
[#] Contributed equally to the manuscript and share first authorship
* Correspondence: sara.witting.christensen.wen@rsyd.dk

Search strings

EMBASE + MEDLINE

Block 1: LUNG CANCER
exp lung cancer/
((lung or pulmonary or bronchial or bronchiogenic or bronchogenic or bronchiolo alveolar or bronchial or non small cell or small cell) adj3 (cancer* or neoplasm* or maligna* or carcinoma* or adenocarcinoma* or squamous cell carcinoma)) or (schneeberg adj3 disease)

Block 2: BLOOD
blood/ or exp arterial blood/ or exp plasma/ or exp serum/
blood or plasma or serum

Block 3: CIRCULATING TUMOR DNA
exp circulating tumor DNA/ or exp liquid biopsy/
((tumor or tumour or cell free or circulating or methylat* or tumor specific or tumour specific) adj3 (dna or gene)) or ctdna or cfdna or liquid biops*

Web of Science

Block 1: LUNG CANCER
(lung cancer*) or (lung neoplasm*) or (lung malignan*) or (lung carcinoma*) or (lung adenocarcinoma*) or (lung squamous cell carcinoma*) or (pulmonary cancer*) or (pulmonary neoplasm*) or (pulmonary malignan*) or (bronchial carcinoma*) or (bronchial adenocarcinoma*) or (bronchial squamous cell carcinoma*) or (bronchial cancer*) or (bronchial neoplasm*) or (bronchial malignan*) or (bronchial carcinoma*) or (bronchial adenocarcinoma*) or (bronchial squamous cell carcinoma*) or (bronchiogenic cancer*) or (bronchiogenic neoplasm*) or (bronchiogenic malignan*) or (bronchiogenic carcinoma*) or (bronchiogenic adenocarcinoma*) or (bronchiogenic squamous cell carcinoma*) or (bronchogenic cancer*) or (bronchogenic neoplasm*) or (bronchogenic malignan*) or (bronchogenic carcinoma*) or (bronchogenic adenocarcinoma*) or (bronchogenic squamous cell carcinoma*) or (bronchiolo alveolar cancer*) or (bronchiolo alveolar neoplasm*) or (bronchiolo alveolar malignan*) or (bronchiolo alveolar carcinoma*) or (bronchiolo alveolar adenocarcinoma*) or (bronchiolo alveolar squamous cell carcinoma*) or (small cell lung cancer*) or (small cell lung neoplasm*) or (small cell lung malignan*) or (small cell lung carcinoma*) or (small cell lung adenocarcinoma*) or (small cell lung squamous cell carcinoma*) or (non small cell lung

cancer*) or (non small cell lung neoplasm*) or (non small cell lung malignan*) or (non small cell lung carcinoma*) or (non small cell lung adenocarcinoma*) or (non small cell lung squamous cell carcinoma*) or (schneeberg disease)

Block 2: BLOOD
blood or plasma or serum

Block 3: CIRCULATING TUMOR DNA
(tumor DNA) or (tumour DNA) or (cell free DNA) or (circulating DNA) or (methylat* DNA) or (tumor specific DNA) or (tumour specific DNA) or ctDNA or cfDNA or (liquid biops*)

Cochrane Library

Block 1: LUNG CANCER
MeSH: lung neoplasms

("lung cancer") or ("lung neoplasm") or ("lung malignancy") or ("lung carcinoma") or ("lung adenocarcinoma") or ("lung squamous cell carcinoma") or ("pulmonary cancer") or ("pulmonary neoplasm") or ("pulmonary malignancy") or ("bronchial carcinoma") or ("bronchial adenocarcinoma") or ("bronchial squamous cell carcinoma") or ("bronchial cancer") or ("bronchial neoplasm") or ("bronchial malignancy") or ("bronchial carcinoma") or ("bronchial adenocarcinoma") or ("bronchial squamous cell carcinoma") or ("bronchiogenic cancer") or ("bronchiogenic neoplasm") or ("bronchiogenic malignancy") or ("bronchiogenic carcinoma") or ("bronchiogenic adenocarcinoma") or ("bronchiogenic squamous cell carcinoma") or ("bronchogenic cancer") or ("bronchogenic neoplasm") or ("bronchogenic malignancy") or ("bronchogenic carcinoma") or ("bronchogenic adenocarcinoma") or ("bronchogenic squamous cell carcinoma") or ("bronchiolo alveolar cancer") or ("bronchiolo alveolar neoplasm") or ("bronchiolo alveolar malignancy") or ("bronchiolo alveolar carcinoma") or ("bronchiolo alveolar adenocarcinoma") or ("bronchiolo alveolar squamous cell carcinoma") or ("small cell lung cancer") or ("small cell lung neoplasm") or ("small cell lung malignancy") or ("small cell lung carcinoma") or ("small cell lung adenocarcinoma") or ("small cell lung squamous cell carcinoma") or ("non small cell lung cancer") or ("non small cell lung neoplasm") or ("non small cell lung malignancy") or ("non small cell lung carcinoma") or ("non small cell lung adenocarcinoma") or ("non small cell lung squamous cell carcinoma") or ("schneeberg disease")

Block 2: BLOOD
MeSH: blood or plasma or serum
blood or plasma or serum

Block 3: CIRCULATING TUMOR DNA
MeSH: circulating tumor DNA or liquid biopsy

("circulating tumor DNA") or ("circulating tumour DNA") or ("cell free DNA") or ("circulating tumor DNA") or ("methylated tumor DNA") or ("tumor specific DNA") or ("tumour specific DNA") or ctDNA or cfDNA or ("liquid biopsy" or "liquid biopsies")

Reviewer agreement
There was good agreement between the reviewers with a proportionate agreement of 0.92 and Cohen's Kappa of 0.74.

Table S1: Comprehensive list of genes

AIM1
CDKN2A
CDO1
DCC
DCLK1
DICER region 1
DLEC1
DROSHA region 1
DROSHA region 2
GATA4
GATA5
HOXA7
HOXA9
KIF1a
KLK10
KMT2C
LINE-1
MGMT
NISCH
p16
PAX5
PCDHGB6
PITX2
PTGDR
PTGER4
RARbeta
RASSF1A
RTEL1
SEPT9
SERPINA1
SFRP1
SHOX2
SHP1P2
SLIT2
SOX17
TAC1
TMPRSS4
UNCX
WT1
ZFP42

Table S2: Contingency tables for all included studies

**Study ID**	**Gene name**	**Test positive Lung cancer positive**	**Test positive Lung cancer negative**	**Test negative Lung cancer positive**	**Test negative Lung cancer negative**
Zeng 2022	SHOX2	74	4	47	117
Ostrow 2009	KIf1a evaluation	3	1	8	23

Ostrow 2009	NISCH evaluation	4	2	7	22
Ostrow 2009	RarB evalutation	5	1	8	23
Ostrow 2009	DCC evaluation	6	0	5	11
Ostrow 2009	B4GALT1 evaluation	1	2	8	8
Ostrow 2009	KIF1A validation	13	1	57	79
Ostrow 2009	DCC validation	19	4	51	76
Ostrow 2009	RARB validation	11	3	59	77
Ostrow 2009	NISCH validation	29	20	41	60
Park 2021	LINE-1	48	8	16	56
Aslam 2017	DCC	19	0	15	34
Usadel 2002	APC serum/plasma	42	0	47	50
Usadel 2002	APC plasma	14	0	1	50
Zhang 2010 A	KLK10	30	2	48	48
Zhang 2010 B	SFRP1	22	2	56	48
Begum 2011	APC training	9	2	16	28
Begum 2011	APC validation	12	3	64	27
Begum 2011	AIM1 traning	4	1	13	14
Begum 2011	AIM1 validation	14	1	62	29
Begum 2011	CDH1 training	10	1	7	14
Begum 2011	CDH1 validation	47	9	29	21
Begum 2011	DCC training	3	0	14	136
Begum 2011	DCC validation	27	0	49	30
Begum 2011	MGMT training	5	1	20	29
Begum 2011	MGMT validation	13	1	63	29
Begum 2011	RASSF1A training	2	1	23	29
Begum 2011	RASSF1A validation	6	1	70	29
Kneip 2011	SHOX2 training	15	1	5	19
Kneip 2011	SHOX2 test	112	16	76	139
Ponomaryova 2011	RARB2	33	13	19	13
Vinayanuwattikun 2011	SHP1P2	34	5	4	47
Gao 2015	RASSF1A plasma benign	25	2	33	29
Gao 2015	RASSF1A plasma healthy		0		23
Gao 2015	RASSF1A serum	21		19	12
Gao 2015	APC plasma benign	14	1	44	30
Gao 2015	APC plasma healthy		0		23
Gao 2015	APC serum	17	2	23	11
Powrozek 2014	SEPT9	31	4	39	96
Balgkouranidou 2014 A	BRMS1 training	23	0	25	24
Balgkouranidou 2014 A	BRMS1 validation	47	0	27	24
Powrozek 2016	DCLK1	32	8	33	87
Balgkouranidou 2016 B	SOX17 training	27	1	21	48

Balgkouranidou 2016 B	SOX17 validation	27	1	47	48
Powrozek 2016	PCDHGB6	29	1	41	79
Powrozek 2016	RTEL1	36	7	34	73
Nunes 2019	APC	32	1	97	27
Nunes 2019	RASSF1A	31	1	98	27
Villalba 2019	TMPRSS4 early (n=16)	14	9	2	16
Hulbert 2017	SOX17	91	8	34	42
Hulbert 2017	TAC1	95	11	30	39
Hulbert 2017	HOXA7	42	4	83	46
Hulbert 2017	CD01	81	13	44	37
Hulbert 2017	HOXA9	108	27	17	23
Hulbert 2017	ZFP42	105	23	20	27
Ooki 2017	HOXA9	12	3	31	39
Ooki 2017	MARCH11	15	6	28	36
Ooki 2017	CDO1	7	2	36	40
Ooki 2017	PTGDR	4	2	39	40
Ooki 2017	UNCX	3	0	40	42
Ooki 2017	AJAP1	2	0	41	42
Yang 2019	CDH13	12	1	27	10
Yang 2019	WT1	12	0	27	11
Yang 2019	CDKN2A	11	0	28	11
Yang 2019	HOXA9	8	0	31	11
Yang 2019	PITX2	11	0	28	11
Yang 2019	CALCA	20	0	19	11
Yang 2019	RASSF1A	16	0	23	11
Yang 2019	DLEC1	16	0	23	11
Li 2020	p16	44	19	4	32
Li 2020	MGMT	45	34	3	17
Li 2020	RASSF1	44	16	4	35
Chen 2020	CDO1	106	17	57	66
Chen 2020	TAC1	110	26	53	57
Chen 2020	SOX17	113	15	50	68
Chen 2020	HOXA7	98	15	65	68
Chen 2020	HOXA9	101	42	62	41
Chen 2020	GATA4	68	35	95	48
Chen 2020	GATA5	72	38	91	45
Chen 2020	PAX5	67	37	96	46
Xu 2020	SHOX2	198	15	104	138
Xu 2020	PTGER4	170	15	132	138
Huang 2020	SHOX2 training	63	1	41	35
Huang 2020	PTGER4 training	56	1	48	35
Huang 2020	SHOX2 validation	12	1	7	10
Huang 2020	PTGER4 validation	10	3	9	9
Mastoraki 2021	KMT2C operable NSCLC	7	0	41	60
Mastoraki 2021	KMT2C stage IV NSCLC	18	0	73	60

Wen 2020	HOXA9	36	2	12	98
Szczyrek 2021	DROSHA region 1	72	11	29	34
Szczyrek 2021	DROSHA region 2	61	7	40	38
Szczyrek 2021	DICER region 1	97	18	4	27
Kim 2022	SLIT2	53	23	19	38
Vo 2022	SHOX2	25	2	5	25
Palanca-Ballester 2022	BCAT1	32	4	12	35
Zhang 2022	SERPINA1 (COPD+LC vs COPD)	12	12	11	44

Table S3: Quality assessment QUADAS-2 – Patient selection

Study ID	Was a consecutive or random sample of patients enrolled?	Was a case-control design avoided?	Did the study avoid inappropriate exclusions?	Could the selection of patients have introduced bias?	Is there concern that the included patients do not match the review question?
Zeng 2022	No	No	Yes	Unclear	Low risk
Ostrow 2009	No	No	Yes	Unclear	Low risk
Park 2021	No	No	Yes	Unclear	Low risk
Aslam 2017	Unclear	No	Yes	Unclear	Low risk
Usadel 2002	No	No	Yes	Unclear	Low risk
Zhang 2010 A	No	No	Yes	Unclear	Low risk
Zhang 2010 B	No	No	Yes	Unclear	Low risk
Begum 2011	Yes	No	Yes	Low risk	Low risk
Kneip 2011	No	No	Yes	High risk	High risk[1]
Ponomaryova 2011	No	No	Yes	Unclear	High risk[2]
Vinayanuwattikun 2011	No	No	Unclear	High risk	Unclear
Gao 2015	Unclear	Yes	Yes	Low risk	Low risk
Powrozek 2014	No	No	Yes	Unclear	Low risk
Balgkouranidou 2014 A	No	No	Yes	High risk	Low risk
Powrozek 2016	No	No	Yes	Unclear	Low risk
Balgkouranidou 2016 B	No	No	Yes	High risk	Low risk
Powrozek 2016	No	No	Yes	Unclear	Low risk
Nunes 2019	No	No	Yes	Unclear	Low risk
Villalba 2019	No	No	Yes	Unclear	Low risk
Hulbert 2017	Unclear	Yes	Yes	Low risk	Low risk
Ooki 2017	No	No	Yes	Unclear	Low risk
Yang 2019	No	Yes	Yes	Low risk	Low risk
Li 2020	No	No	Yes	Unclear	Low risk
Chen 2020	Unclear	Yes	Yes	Low risk	Low risk
Xu 2020	No	No	Yes	Unclear	Low risk
Huang 2020	Yes	No	Yes	Low risk	Low risk
Mastoraki 2021	No	No	Yes	Low risk	Low risk

Wen 2020	No	No	Yes	High risk	High risk[3]
Szczyrek 2021	Yes	No	Yes	Low risk	Low risk
Kim 2022	Unclear	No	Yes	Unclear	Low risk
Vo 2022	No	No	Yes	Unclear	Low risk
Palanca-Ballester 2022	No	No	Yes	Unclear	Low risk
Zhang 2022	Yes	No	Yes	Low risk	Low risk

Patient selection: Is there concern that the included patients do not match the review question? Supporting text:
1: Because there are many cases and controls with missing data. Half of the cases and controls have no reported age, 20-25% have no reported gender, 55% of cases have other or unknown lung cancer histology, and 4 controls have prostate cancer.
2: Because the control group consisted entirely of males.
3: Only adenocarcinoma patients.

Table S4: Quality assessment QUADAS-2 – Index test

Study ID	Were the index test results interpreted without knowledge of the results of the reference standard?	If a threshold was used, was it pre-specified?	Could the conduct or interpretation of the index test have introduced bias?	Is there concern that the index test, its conduct, or interpretation differ from the review question?
Zeng 2022	Unclear	No	Unclear	Low risk
Ostrow 2009	Unclear	Yes	Low risk	Low risk
Park 2021	Yes	No	Low risk	Low risk
Aslam 2017	Unclear	Unclear	Unclear	Low risk
Usadel 2002	Unclear	Unclear	Unclear	Low risk
Zhang 2010 A	Unclear	Unclear	Unclear	Low risk
Zhang 2010 B	Unclear	Unclear	Unclear	Low risk
Begum 2011	Unclear	Yes	Low risk	Low risk
Kneip 2011	Unclear	No	Unclear	Low risk
Ponomaryova 2011	Unclear	No	Unclear	Low risk
Vinayanuwattikun 2011	Unclear	No[2]	Unclear	High risk
Gao 2015	Unclear	Unclear	Unclear	Low risk
Powrozek 2014	Unclear	Yes	Low risk	Low risk
Balgkouranidou 2014 A	No	Unclear	Low risk	Low risk
Powrozek 2016	Unclear	Unclear	Unclear	Low risk
Balgkouranidou 2016 B	No	Unclear	Low risk	Low risk
Powrozek 2016	Unclear	No	Unclear	Low risk
Nunes 2019	Unclear	Yes	Low risk	Low risk
Villalba 2019	Unclear	No	Unclear	Low risk
Hulbert 2017	Unclear	No	Unclear	Low risk
Ooki 2017	Unclear	Yes	Low risk	Low risk

Yang 2019	Unclear	No	Low risk	Low risk
Li 2020	Unclear	No[3]	Unclear	Low risk
Chen 2020	Unclear	No	Unclear	Low risk
Xu 2020	Yes	Yes	Low risk	Low risk
Huang 2020	Unclear	Yes	Low risk	Low risk
Mastoraki 2021	Unclear	Yes	Low risk	Low risk
Wen 2020	Unclear	Yes	Low risk	Low risk
Szczyrek 2021	Unclear	Unclear	Unclear	Low risk
Kim 2022	Unclear	Yes	Low risk	Low risk
Vo 2022	Unclear	No[4]	Unclear	Low risk
Palanca-Ballester 2022	Unclear	No	Unclear	Low risk
Zhang 2022	Unclear	Unclear	Unclear	Low risk

If a threshold was used, was it pre-specified? Supporting text:
1: Not quantitative but verified by sequencing.
2: Only ROC analysis of the cohort in the study.
3: Cutoff set in the present study.
4: Only training cohort, no validation cohort.

Table S5: Quality assessment QUADAS-2 – Reference standard

**Study ID**	**Is the reference standard likely to correctly classify the target condition?**	**Were the reference standard results interpreted without knowledge of the results of the index test?**	**Could the reference standard, its conduct, or its interpretation have introduced bias?**	**Is there concern that the target condition as defined by the reference standard does not match the review question?**
Zeng 2022	Yes	Yes	Low risk	Low risk
Ostrow 2009	Yes	Yes	Low risk	Low risk
Park 2021	Yes	Yes	Low risk	Low risk
Aslam 2017	Yes	Yes	Low risk	Low risk
Usadel 2002	Yes	Yes	Low risk	Low risk
Zhang 2010 A	Yes	Yes	Low risk	Low risk
Zhang 2010 B	Yes	Yes	Low risk	Low risk
Begum 2011	Yes	Yes	Low risk	Low risk
Kneip 2011	Unclear[1]	Yes	Unclear	Unclear
Ponomaryova 2011	Yes	Yes	Low risk	Low risk
Vinayanuwattikun 2011	Yes	Yes	Low risk	Low risk
Gao 2015	Yes	Yes	Low risk	Low risk
Powrozek 2014	Yes	Yes	Low risk	Low risk
Balgkouranidou 2014 A	Yes	Yes	Low risk	Low risk
Powrozek 2016	Yes	Yes	Low risk	Low risk
Balgkouranidou 2016 B	Yes	Yes	Low risk	Low risk

Powrozek 2016	Yes	Yes	Low risk	Low risk
Nunes 2019	Yes	Yes	Low risk	Low risk
Villalba 2019	Yes	Yes	Low risk	Low risk
Hulbert 2017	Yes	Yes	Low risk	Low risk
Ooki 2017	Yes	Yes	Low risk	Low risk
Yang 2019	Yes	Yes	Low risk	Low risk
Li 2020	Yes	Yes	Low risk	Low risk
Chen 2020	Yes	Yes	Low risk	Low risk
Xu 2020	Yes	Yes	Low risk	Low risk
Huang 2020	Yes	Yes	Low risk	Low risk
Mastoraki 2021	Yes	Yes	Low risk	Low risk
Wen 2020	Yes	Yes	Low risk	Low risk
Szczyrek 2021	Yes	Yes	Low risk	Low risk
Kim 2022	Yes	Yes	Low risk	Low risk
Vo 2022	Yes	Yes	Low risk	Low risk
Palanca-Ballester 2022	Yes	Yes	Low risk	Low risk
Zhang 2022	Yes	Yes	Low risk	Low risk

Is the reference standard likely to correctly classify the target condition? Supporting text:
1: The choice of reference standard was not described.

Table S6: Quality assessment QUADAS-2 – Flow and timing

Study ID	Was there an appropriate interval between index test and reference standard?	Did all patients receive a reference standard?	Did patients receive the same reference standard?	Were all patients included in the analysis?	Could the patient flow have introduced bias?
Zeng 2022	Yes	Yes	Yes	Yes	Low risk
Ostrow 2009	Unclear	Yes	Yes	Yes	Low risk
Park 2021	Unclear	Yes	Yes	Yes	Unclear
Aslam 2017	Unclear	Yes	Yes	Yes	Low risk
Usadel 2002	Yes	Yes	Yes	Yes	Low risk
Zhang 2010 A	Yes	Yes	Yes	Yes	Low risk
Zhang 2010 B	Yes	Yes	Yes	Yes	Low risk
Begum 2011	Unclear	Yes	Yes	Yes	Low risk
Kneip 2011	Unclear	Unclear	Unclear	No	Unclear
Ponomaryova 2011	Unclear	Yes	Yes	Yes	Low risk
Vinayanuwattikun 2011	Unclear	Yes	Yes	Yes	Unclear
Gao 2015	Unclear	Yes	Yes	Yes	Low risk
Powrozek 2014	Unclear	Yes	Yes	Yes	Low risk
Balgkouranidou 2014 A	Unclear	Yes	No[3]	Yes	Low risk
Powrozek 2016	Yes	Yes	Yes	Yes	Low risk
Balgkouranidou 2016 B	Unclear	Yes	No	Yes	Low risk

Powrozek 2016	Yes	Yes	Yes	Yes	Low risk
Nunes 2019	Yes	Yes	Yes	Yes	Low risk
Villalba 2019	Unclear	Yes	Yes	Yes	Unclear
Hulbert 2017	No[1]	Yes	Yes	No	Low risk
Ooki 2017	Unclear	Yes	Yes	Unclear	Unclear
Yang 2019	Yes	Yes	Yes	Yes	Low risk
Li 2020	Unclear	Yes	Yes	Yes	Low risk
Chen 2020	Yes	Yes	Yes	Yes	Low risk
Xu 2020	Unclear	Yes	Yes	Yes	Low risk
Huang 2020	Yes	Yes	Yes	Yes	Low risk
Mastoraki 2021	No[2]	Yes	Yes	Yes	Low risk
Wen 2020	Unclear	Yes	Yes	Yes	Low risk
Szczyrek 2021	Unclear	Yes	Yes	Yes	Low risk
Kim 2022	No	Yes	Unclear	No	High risk
Vo 2022	Yes	Yes	Yes	Yes	Unclear
Palanca-Ballester 2022	Unclear	Yes	Yes	Yes	Low risk
Zhang 2022	Yes	Yes	Yes	Yes	Low risk

Was there an appropriate interval between index test(s) and reference standard? Supporting text:
1: Too long time plasma storage.
2: Too long. Samples were collected in 2004-2006. Not stated when they were analyzed, but study published in 2021.

Did patients receive the same reference standard? Supporting text:
3: Some had surgery to confirm the diagnosis, others had biopsies.

Table S7: Quality assessment – Funding and conflicts of interest

Study ID	Funding: How was the study funded?	Conflicts of interest
Zeng 2022	Public/non-profit funding sources	None
Ostrow 2009	Company/corporate funding sources	Yes, pertaining to the funding sources
Park 2021	Public/non-profit funding sources	None
Aslam 2017	Public/non-profit funding sources	None
Usadel 2002	Public/non-profit funding sources	Yes, but not pertaining to the funding sources
Zhang 2010 A	Unclear	Not stated
Zhang 2010 B	Unclear	Not stated
Begum 2011	Company/corporate funding sources	Yes, pertaining to the funding sources
Kneip 2011	Unclear	Yes, pertaining to the funding sources
Ponomaryova 2011	Public/non-profit funding sources	None
Vinayanuwattikun 2011	Public/non-profit funding sources	None
Gao 2015	Public/non-profit funding sources	None
Powrozek 2014	Unclear	None
Balgkouranidou 2014 A	Public/non-profit funding sources	Not stated
Powrozek 2016	Company/corporate funding sources	None

Balgkouranidou 2016 B	Public/non-profit funding sources	None
Powrozek 2016	Company/corporate funding sources	None
Nunes 2019	Public/non-profit funding sources	None
Villalba 2019	Public/non-profit funding sources	None
Hulbert 2017	Public/non-profit funding sources	Yes, but not pertaining to the funding sources
Ooki 2017	Public/non-profit funding sources	None
Yang 2019	Public/non-profit funding sources	None
Li 2020	Public/non-profit funding sources	None
Chen 2020	Public/non-profit funding sources	None
Xu 2020	Public/non-profit funding sources	None
Huang 2020	Public/non-profit funding sources	None
Mastoraki 2021	Public/non-profit funding sources	None
Wen 2020	Public/non-profit funding sources	Yes, but not pertaining to the funding sources
Szczyrek 2021	Public/non-profit funding sources	None
Kim 2022	Public/non-profit funding sources	None
Vo 2022	Public/non-profit funding sources	None
Palanca-Ballester 2022	Public/non-profit funding sources	None
Zhang 2022	Public/non-profit funding sources	None

Risk of publication bias

Suspicion of publication bias can arise when a higher proportion of small studies report bigger effect sizes compared to larger studies. The risk of publication bias in the current review was visualized by Deek's Funnel Plot (Figure S1). The asymmetry test showed no statistically significant publication bias (p=0.30).

Figure S1: Funnel Plot

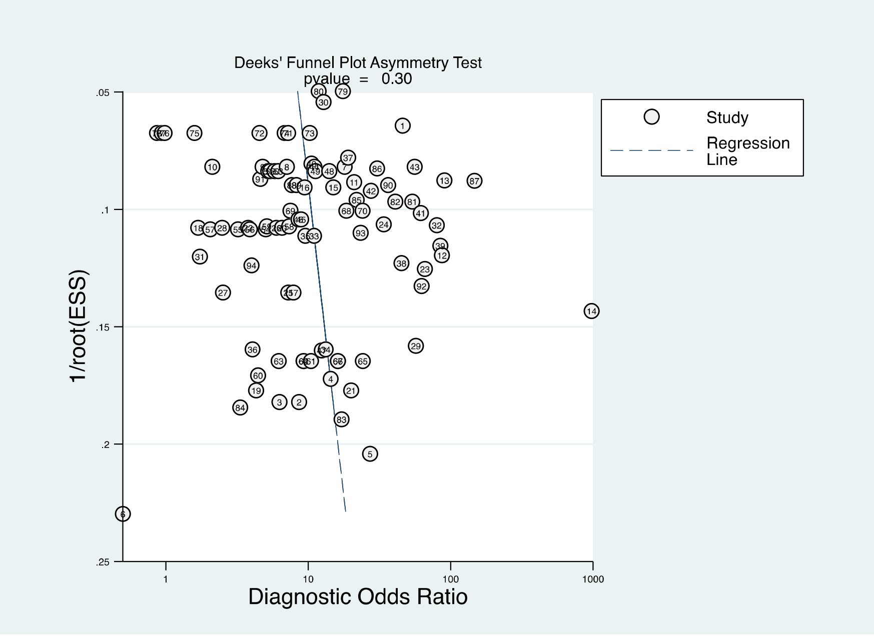

Supplement: Supplementary file 1 [file cancers-15-03959-s001.zip › cancers-2516903-supplementary.pdf]
